# Supplementary material for: Biochemical and Initial Structural Characterization of the Monocot Chimeric Jacalin OsJAC1
Source: Int J Mol Sci. 2021 May 26;22(11):5639. doi: 10.3390/ijms22115639 (PMC8197871; doi:10.3390/ijms22115639)
Supplement: Supplementary file 1 [file ijms-22-05639-s001.zip › ijms-1208848-supplementary.pdf]

Figure S1: SDS-PAGE (12%) of DIR domain protein heterologously produced in *E. coli* BL21(DE3) and isolated by IMAC. S2
Figure S2: Size-exclusion chromatogram of OsJAC1 without DTT (blue) and with 1 mM DTT (red). S2
Figure S3: Histogram (left) and radius plot (right) of OsJAC1 in 15 mM TRIS-HCl (pH 7.4) determined by DLS. S3
Figure S4: Histogram (left) and radius plot (right) of OsJAC1 in 15 mM TRIS-HCl (pH 7.4) and 150 mM $CaCl_2$ determined by DLS. S3
Figure S5: Histogram (left) and radius plot (right) of OsJAC1 in 15 mM TRIS-HCl (pH 7.4) and 50 mM lactose determined by DLS. S4
Figure S6: Histogram (left) and radius plot (right) of OsJAC1 in 15 mM TRIS-HCl (pH 7.4) and 150 mM $MgSO_4$ determined by DLS. S4
Figure S7: Histogram plot of OsJAC1 in 15 mM TRIS-HCl (pH 7.4) and 4 mM DTT determined by DLS. S5
Figure S8: Negative test of the interaction study. Melting point (T_M) of BSA and lysozym in relation to different saccharides (50 mM). S5
Figure S9: Melting point (T_M) of the prominent transition above 70°C for OsJAC1 in relation to different saccharides (50 mM) and in the presence of 4 mM DTT. S6
Figure S10: Alignment of the OsJAC1 domain JRL and Banlec from the banana fruit (*Musa acuminata*; pdb code: 4PIF) sequence. Purple shading indicates identical amino acids. Binding site one of Banlec is framed red and binding site two of Banlec is framed black[1]. Sequences were aligned by using Clustal Omega[2]. S8
Figure 11: Savitzky-Golay-smoothed[16] near-UV CD spectra of the OsJAC1 domain DIR alone and in the presence of 1 mM galactobiose or laminaribiose (50 mM KPi buffer, pH 7.4). S8
Figure 12: Savitzky-Golay-smoothed[16] near-UV CD spectra of the OsJAC1 domain JRL alone and in the presence of 1 mM galactobiose or laminaribiose (50 mM KPi buffer, pH 7.4). S9
Figure 13: Savitzky-Golay-smoothed[16] near-UV CD spectra of OsJAC1 alone and in the presence of 1 mM galactobiose or laminaribiose (50 mM KPi buffer, pH 7.4). S9
Table S1: Melting point (T_M) of the prominent transition above 70°C for OsJAC1 and its two domain proteins JRL and DIR in relation to different saccharides (50 mM) without DTT. S8
Table S2: Melting point (T_M) of both transition points for OsJAC1 and mixture of both domain protein JRL and DIR (1:1) in relation to different saccharides (50 mM) with 4 mM DTT. S8

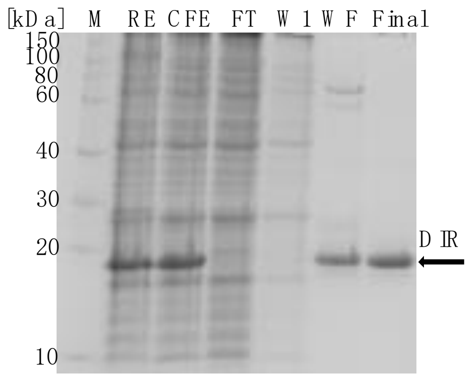

Figure S1: SDS-PAGE (12%) of DIR domain protein heterologously produced in *E. coli* BL21(DE3) and isolated by IMAC.

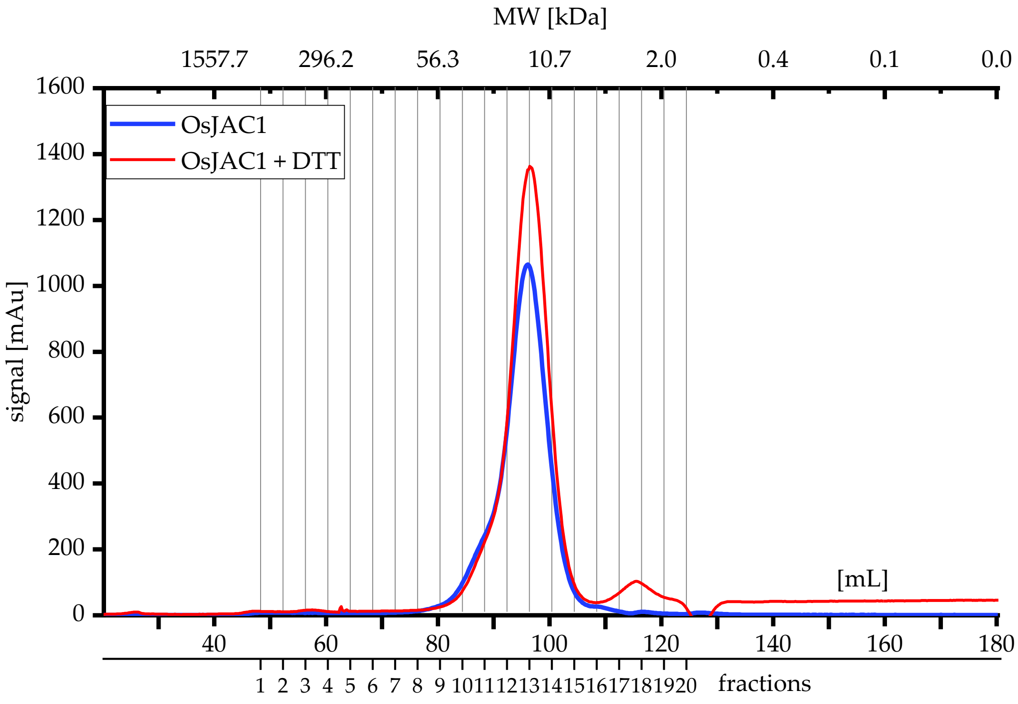

Figure S2: Size-exclusion chromatogram of OsJAC1 without DTT (blue) and with 1 mM DTT (red).

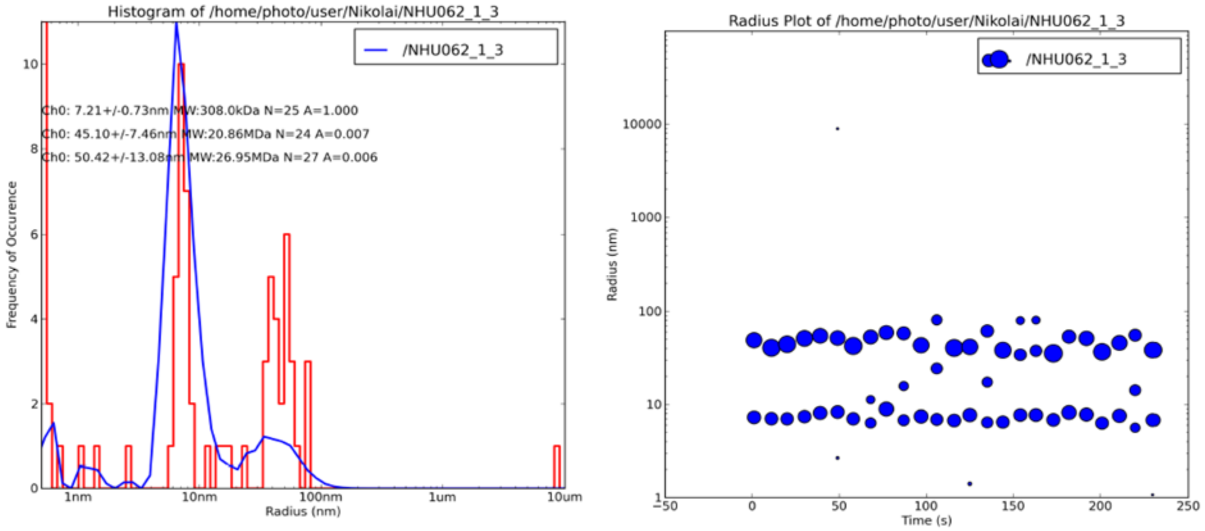

Figure S3: Histogram (left) and radius plot (right) of OsJAC1 in 15 mM TRIS-HCl (pH 7.4) determined by DLS.

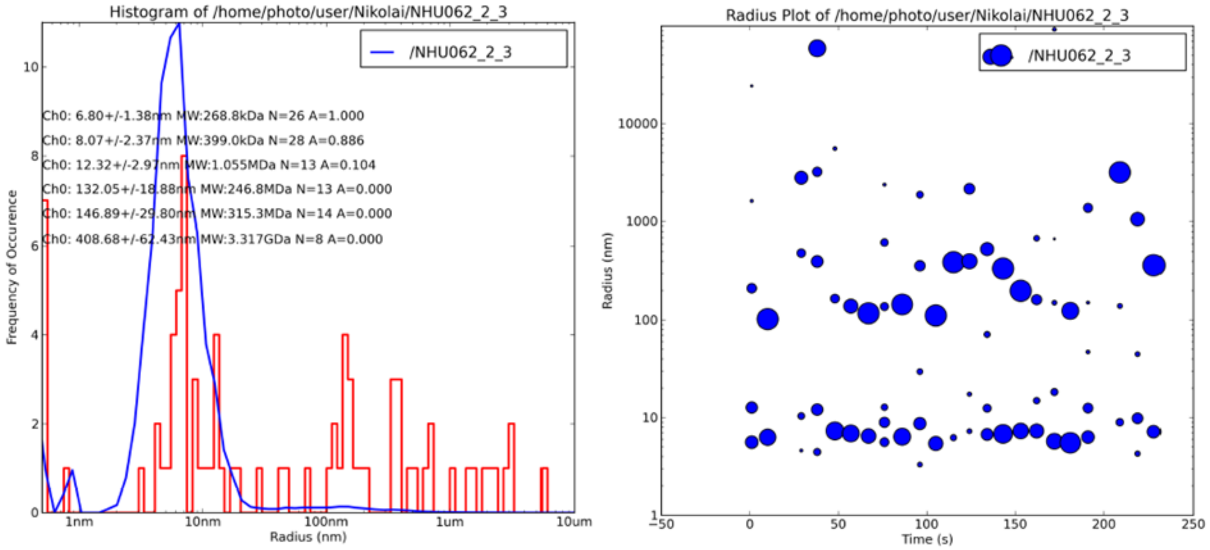

Figure S4: Histogram (left) and radius plot (right) of OsJAC1 in 15 mM TRIS-HCl (pH 7.4) and 150 mM $CaCl_2$ determined by DLS.

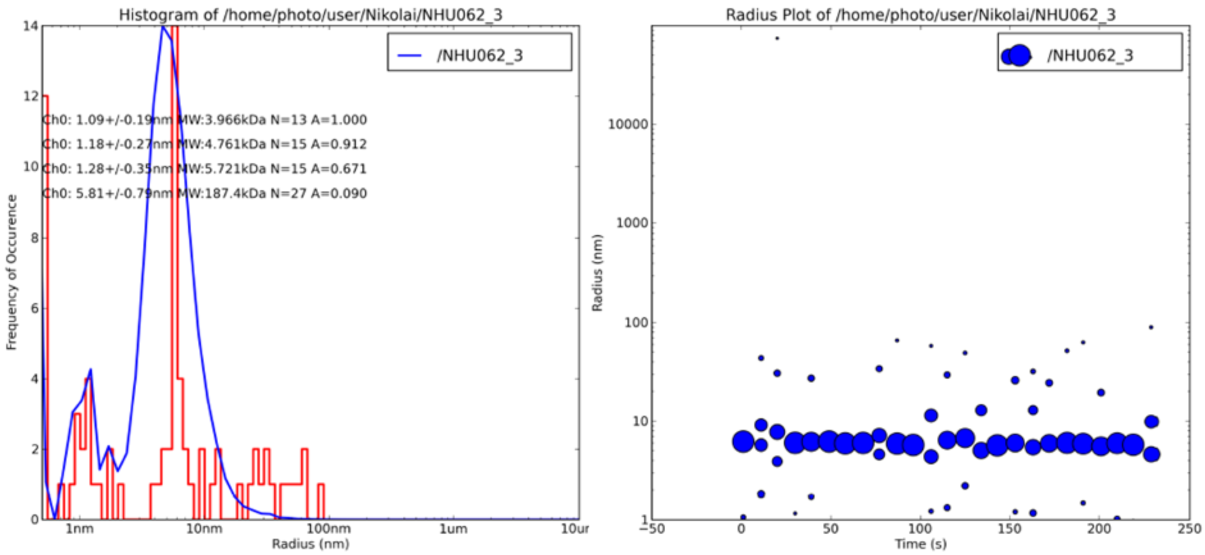

Figure S5: Histogram (left) and radius plot (right) of OsJAC1 in 15 mM TRIS-HCl (pH 7.4) and 50 mM lactose determined by DLS.

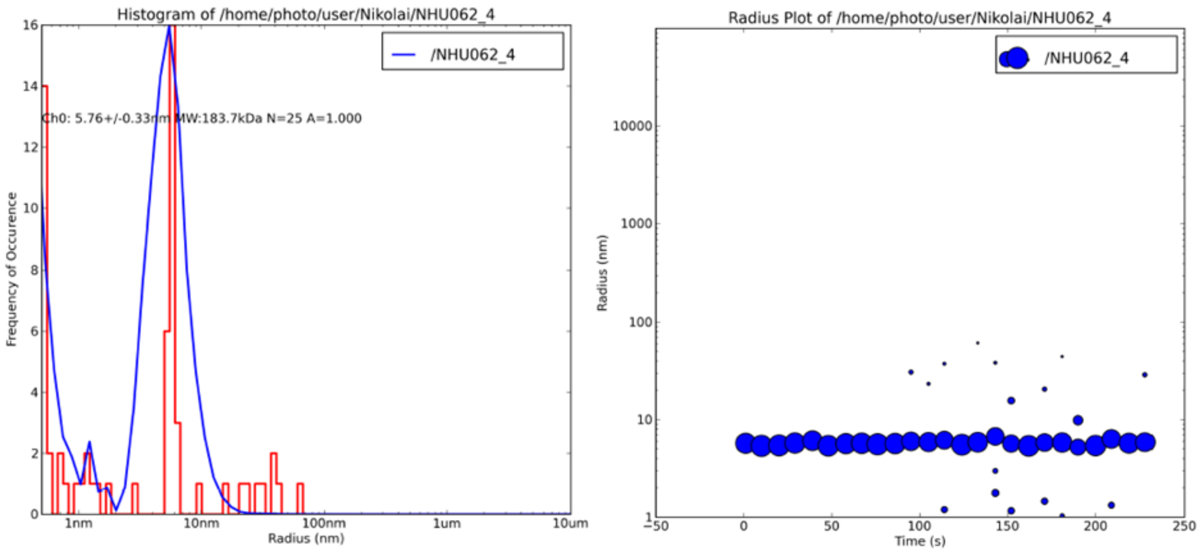

Figure S6: Histogram (left) and radius plot (right) of OsJAC1 in 15 mM TRIS-HCl (pH 7.4) and 150 mM $MgSO_4$ determined by DLS.

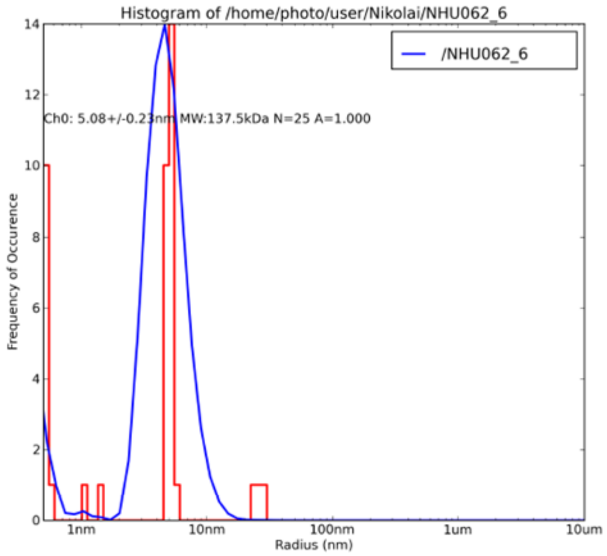

Figure S7: Histogram plot of OsJAC1 in 15 mM TRIS-HCl (pH 7.4) and 4 mM DTT determined by DLS.

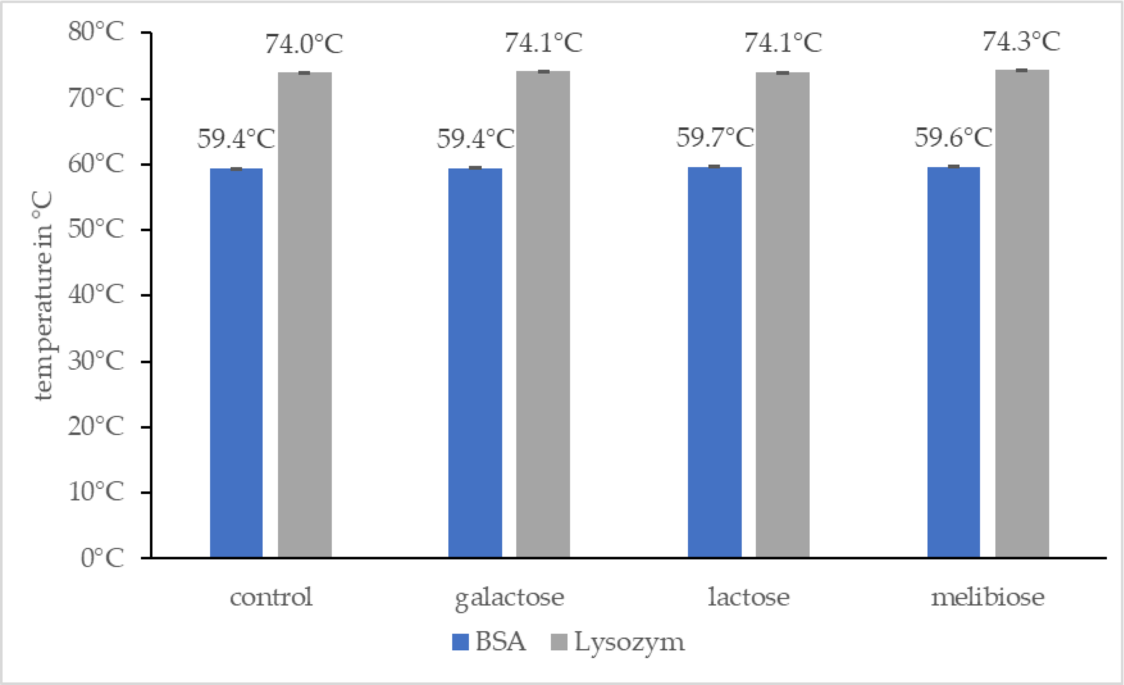

Figure S8: Negative test of the interaction study. Melting point (T_M) of BSA and lysozym in relation to different saccharides (50 mM).

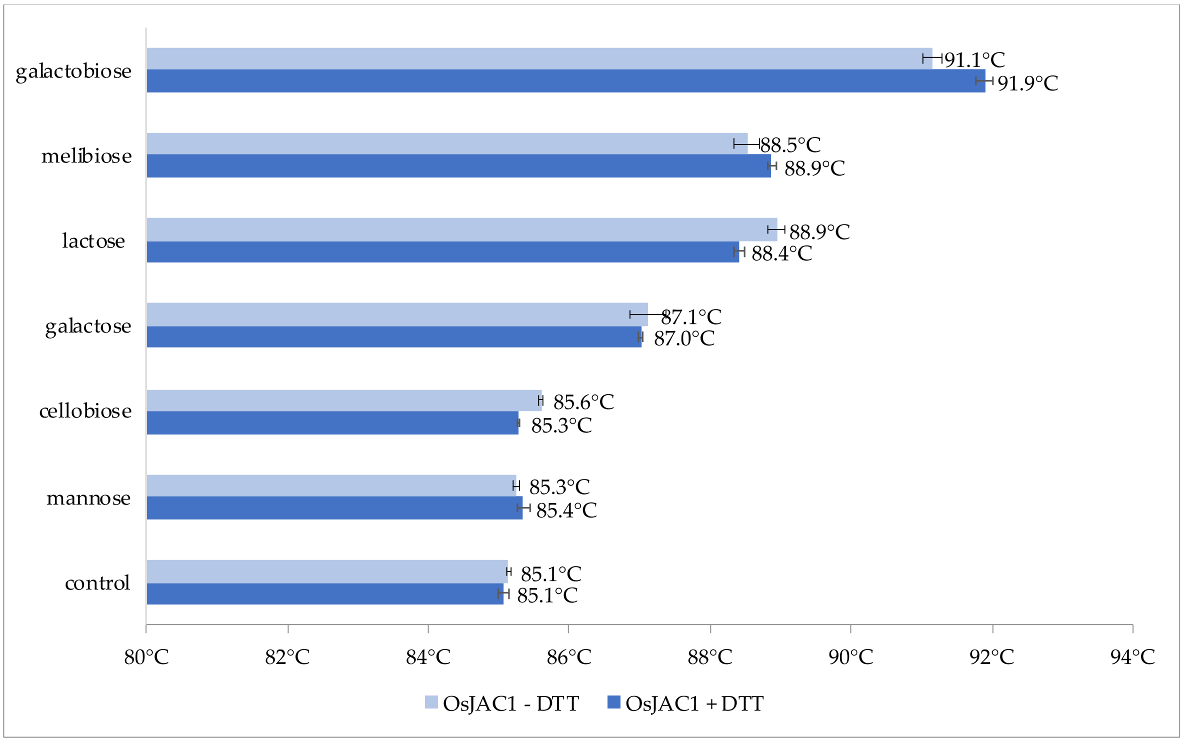

Figure S9: Melting point (T_M) of the prominent transition above 70°C for OsJAC1 in relation to different saccharides (50 mM) and in the presence of 4 mM DTT.

Table S1: Melting point (T_M) of the prominent transition above 70°C for OsJAC1 and its two domain proteins JRL and DIR in relation to different saccharides (50 mM) without DTT.

without DTT	OsJAC1 T_{M2} (°C)	±	JRL T_M (°C)	±	DIR T_M (°C)	±
control	85.1°C	0.05	73.7°C - 74.2°C	0.05	77.4°C	0.02
Sugar						
D-(+)-glucose	85.4°C	0.05	74.9°C	0.05	77.8°C	0.05
D-(+)-mannose	85.3°C	0.04	75.2°C	0.04	77.7°C	0.05
L-(+)-rhamnose	85.0°C	0.15	74.4°C	0.03		
D-sorbitol	85.4°C	0.04	74.4°C	0.06		
D-(+)-xylose	85.1°C	0.04	74.6°C	0.04		
N-acetyl-β-D-glucosamin	85.6°C	0.06	74.9°C	0.07		
N-acetyl-D-galactosamin	85.6°C	0.17	74.3°C	0.06	78.7°C	0.17
D-mannitol	85.7°C	0.15				
D-(+)-galactose	87.1°C	0.25	74.5°C	0.09	80.6°C	0.04
lactose	88.9°C	0.13	74.6°C	0.05	82.5°C	0.07
D-(+)-cellobiose	85.6°C	0.04	74.1°C	0.03	78.0°C	0.03
D-(+)-melibiose	88.5°C	0.18	74.3°C	0.06	82.8°C	0.19
N,N'-diacetyl-chitobiose	85.8°C	0.18	74.5°C	0.20	77.9°C	0.11
laminaribiose	84.7°C	0.01	75.9°C	0.03	77.8°C	0.33
1,4-β-D-galactobiose	91.1°C	0.12	74.3°C	0.12	80.0°C	0.16
galactan	84.7°C	0.05	74.3°C	0.12	77.4°C	0.10
maltose	85.6°C	0.04	74.7°C	0.06		
sucrose	85.7°C	0.12	74.6°C	0.06		
1,2-α-mannobiose	86.3°C	0.16				

Table S2: Melting points (T_M) of OsJAC1 and DIR/JRL (1:1) protein mixture in relation to different saccharides (50 mM) with 4 mM DTT.

with DTT	OsJAC1 T_{M1} (°C)	±	T_{M2} (°C)	±	JRL & DIR T_{M1} (°C)	±	T_{M2} (°C)	±
control	60.7°C	0.08	85.7°C	0.11	59.4°C	0.14	77.7°C	0.03
Sugar								
D-(+)-glucose	61.8°C	0.19	85.8°C	0.24	60.4°C	0.02	77.9°C	0.02
D-(+)-mannose	61.8°C	0.14	85.6°C	0.09	60.8°C	0.10	78.0°C	0.04
N-acetyl-β-D-glucosamin					60.0°C	0.09	78.0°C	0.07
N-acetyl-D-galactosamin					59.6°C	0.03	78.5°C	0.03
D-(+)-galactose	61.2°C	0.08	87.6°C	0.08	59.7°C	0.05	80.6°C	0.07
lactose	61.0°C	0.02	88.7°C	0.02	59.7°C	0.01	81.9°C	0.02
D-(+)-cellobiose	60.9°C	0.00	85.2°C	0.13	59.9°C	0.19	77.9°C	0.09
D-(+)-melibiose	61.1°C	0.00	88.8°C	0.07	59.8°C	0.17	82.4°C	0.13
N,N'-diacetylchitobiose					59.6°C	0.08	78.0°C	0.01
laminaribiose	63.3°C	0.08	86.3°C	0.02	61.6°C	0.06	78.5°C	0.01
1,4-β-D-galactobiose	61.1°C	0.06	91.9°C	0.12	59.7°C	0.07	85.8°C	0.06
galactan					59.2°C	0.03	78.0°C	0.01
1,2-α-mannobiose	62.6°C	0.08	85.8°C	0.09	61.2°C	0.19	78.2°C	0.20

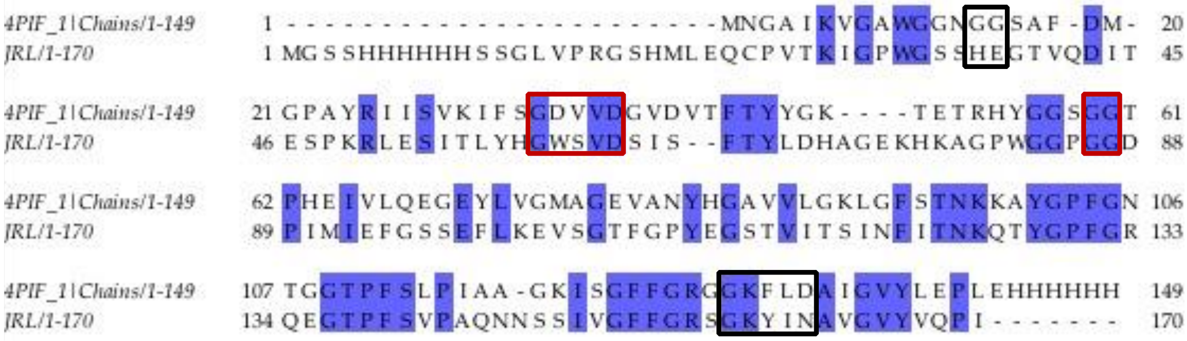

Figure S10: Alignment of the OsJAC1 domain JRL and Banlec from the banana fruit (*Musa acuminata*; pdb code: 4PIF) sequence. Purple shading indicates identical amino acids. Binding site one of Banlec is framed red and binding site two of Banlec is framed black[1]. Sequences were aligned by using Clustal Omega[2].

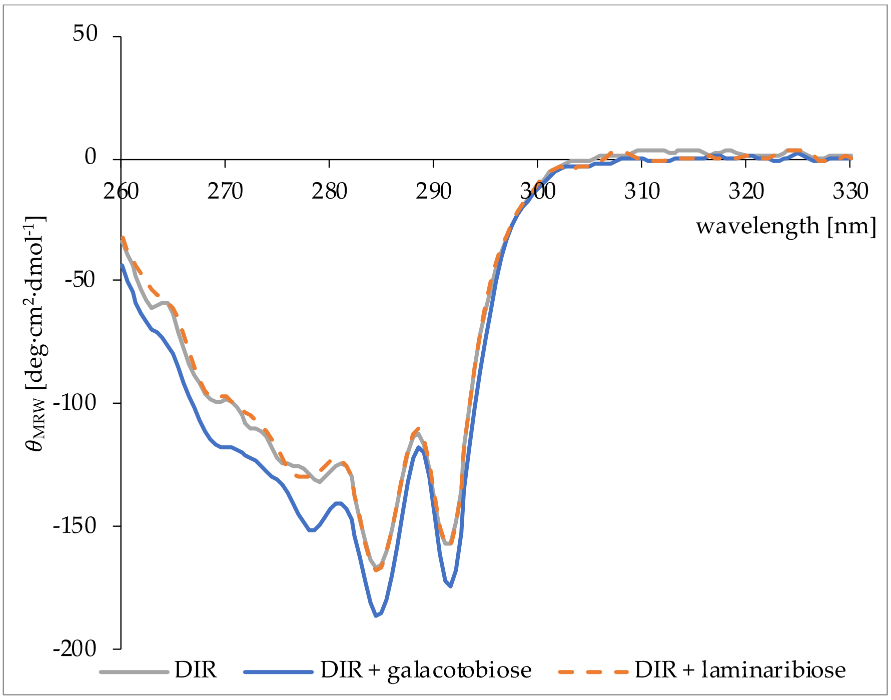

Figure 11: Savitzky-Golay-smoothed[16] near-UV CD spectra of the OsJAC1 domain DIR alone and in the presence of 1 mM galactobiose or laminaribiose (50 mM KPi buffer, pH 7.4).

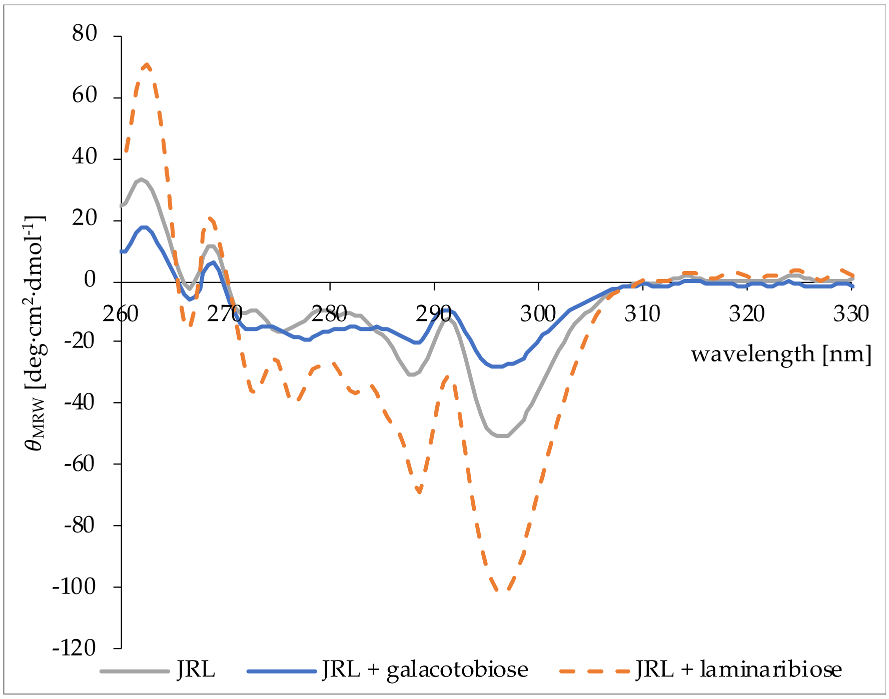

Figure 12: Savitzky-Golay-smoothed[16] near-UV CD spectra of the OsJAC1 domain JRL alone and in the presence of 1 mM galactobiose or laminaribiose (50 mM KPi buffer, pH 7.4).

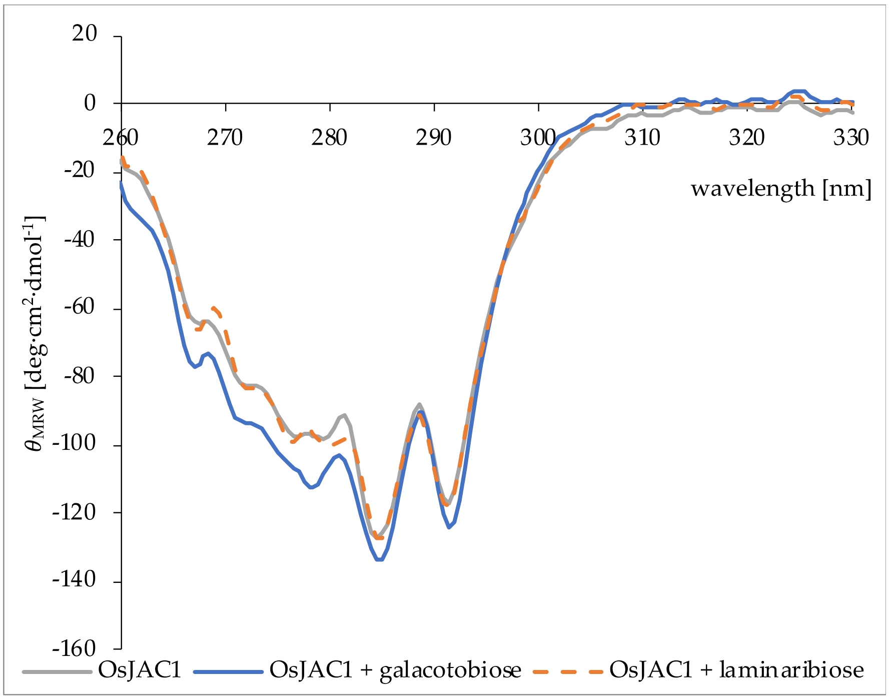

Figure 13: Savitzky-Golay-smoothed[16] near-UV CD spectra of OsJAC1 alone and in the presence of 1 mM galactobiose or laminaribiose (50 mM KPi buffer, pH 7.4).

Supplemental References

1. Meagher, J.L.; Winter, H.C.; Ezell, P.; Goldstein, I.J.; Stuckey, J.A. Crystal structure of banana lectin reveals a novel second sugar binding site. *Glycobiology* **2005**, *15*, 1033-1042.
2. Sievers, F.; Wilm, A.; Dineen, D.; Gibson, T.J.; Karplus, K.; Li, W.; Lopez, R.; McWilliam, H.; Remmert, M.; Söding, J. Fast, scalable generation of high-quality protein multiple sequence alignments using Clustal Omega. *Molecular systems biology* **2011**, *7*, 539.

© 2020 by the authors. Submitted for possible open access publication under the terms and conditions of the Creative Commons Attribution (CC BY) license (http://creativecommons.org/licenses/by/4.0/).
